# Understanding the Determinants of Adolescent Pregnancy among an Indigenous Community in Rural Nepal: A Qualitative Exploration

**DOI:** 10.1002/puh2.70041

**Published:** 2025-03-11

**Authors:** Kusumsheela Bhatta, Pratiksha Pathak, Madhusudan Subedi

**Affiliations:** ^1^ School of Public Health Patan Academy of Health Sciences Lalitpur Nepal

**Keywords:** adolescent | Chepang | determinants | Nepal | pregnancy | qualitative research

## Abstract

**Introduction:**

Adolescent pregnancy is a global concern, with a higher occurrence among more disadvantaged groups. Within Nepal's highly marginalized Chepang community, adolescent pregnancies are notably more prevalent. This study aimed to understand the determinants of adolescent pregnancy among Chepang community in Raksirang Rural Municipality, Makwanpur District of Nepal using qualitative methods.

**Methods:**

The study was conducted from September 2022 to April 2023. Twenty participants (10 in‐depth interviews and 10 key informant interviews) were recruited through judgmental sampling. An interview guide was used for interviewing the participants. All interviews were audio‐recorded, transcribed, translated, and analyzed using R‐based Qualitative Data Analysis package. Braun and Clarke's six‐step thematic analysis was used to perform analysis.

**Results:**

Six key themes were identified as determinants of adolescent pregnancy among Chepang community: (i) inadequate access to and use of sexual and reproductive health services, (ii) gaps in programmatic implementation, (iii) elopement marriage as a form of escape, (iv) discontinuation of education, (v) limited knowledge and understanding of marriage, pregnancy, and childbirth, and (vi) early pregnancy as a sign of prosperity. Challenges such as difficult geographical terrain, limited access to adolescent‐friendly healthcare, gender inequality, child labor, weak enforcement of child marriage laws, and societal stigma exacerbated these determinants.

**Conclusion:**

A multifaceted approach, including improved access to adolescent‐friendly healthcare, enforcement of child marriage laws, access to secondary education, efforts to raise awareness of sexual and reproductive health, and a shift in sociocultural norms, should be implemented to reduce adolescent pregnancy in the Chepang community.

## Graphical abstract

Adolescent pregnancy remains a critical issue in Nepal's marginalized Chepang community. Our study identified key determinants, including limited access to sexual and reproductive health services, elopement marriages, school dropouts, poverty, socio‐cultural norms, fears about post‐contraception fertility, weak enforcement of child marriage laws, and a lack of awareness.

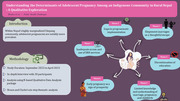

## 1 Introduction

Adolescence, as defined by the World Health Organization (WHO), refers to the period from ages 10 to 19, representing the transition from childhood to adulthood [1]. Adolescent pregnancy is a global public health concern affecting high‐, middle‐, and low‐income countries, with higher prevalence in disadvantaged communities due to early marriage, poverty, limited access to education and employment opportunities, and limited availability and utilization of healthcare services [2, 3]. Pregnancies in girls under 19 have lasting impacts and can compromise their rights, posing life‐threatening risks to their developmental, sexual, and reproductive health [4, 5]. In Nepal after declining between 2001 (21%) and 2011 (17%), adolescent pregnancy remained constant from 2011 to 2016 (17%) [6]. As per the Nepal Demographic Health Survey (NDHS) 2022, adolescent pregnancy has declined to 14%. However, the high prevalence of child marriage, with 21% of women aged 15–19 currently married, remains a significant concern and a major determinant of adolescent pregnancy [7].

The Interim Constitution of Nepal states that every woman has the right to reproductive health and other related matters [8]. This includes access to healthcare services, information, education, and protection from abuse and coercion, among other rights [9]. The National Penal Code of 2017 criminalizes child marriage, setting the eligible age for marriage at 20, with violations punishable by imprisonment and fines [10]. Further, the Nepal government adopted the Adolescent Development and Health Strategy in 2002 followed by the National Adolescent and Sexual Reproductive Health (ASRH) in 2011 [11].

The Chepang community is one of the highly marginalized groups living on the hilly and steeper slopes of primarily Chitwan, Dhading, Gorkha, and Makwanpur District of Nepal [12]. The Chepang community in Nepal, comprising 84,364 individuals, constitutes approximately 0.29% of the total national population [13]. A community‐based cross‐sectional study reported an adolescent pregnancy prevalence of 71.4% among Chepang women and found that 72.7% were married before the age of 18 [14].

Existing research on adolescent pregnancy in Nepal primarily focuses on hospital‐based studies and neglects community‐based factors such as family and societal norms, law enforcement, and access to reproductive health services [15]. This study aimed to provide a comprehensive picture of the multidimensional determinants of adolescent pregnancy among the Chepang communities of Raksirang Rural Municipality through qualitative inquiry.

## 2 Methods

### 2.1 Study Design and Setting

This study used an exploratory inductive qualitative design to understand the determinants of adolescent pregnancy among Chepangs. The study was conducted in Wards 5 and 8 of Raksirang Rural Municipality, Makwanpur District. Both wards were selected due to their significant population of Chepang indigenous community members, allowing us to delve into the specific socio‐cultural dynamics influencing adolescent pregnancy within this demographic. The study duration was for 12 months starting from September 2022 to April 2023.

### 2.2 Participants and Recruitment

On the basis of Warren's suggestion regarding the minimum number of interviews in the qualitative study, a total of 25 interviews were proposed [16]. Out of the anticipated 15 in‐depth interviews (IDIs) with Chepang mothers, the interview was stopped after the 10th IDI due to data saturation as no new information was coming in terms of factors influencing adolescent pregnancy. Likewise, the key informant's interviews (KIIs) were done with four female community health volunteers (FCHVs), one health coordinator, two elected political leaders, one school teacher, and two other health care providers (Table 1).

**TABLE 1:** Participants for the study.

Type of study participants	Proposed number of interviews	Number of interviews taken	Method
Chepang mothers	15	10	IDI
FCHV	4	4	KII
Health coordinator	1	1	KII
Elected political leaders	2	2	KII
School teacher	1	1	KII
Other healthcare providers	2	2	KII

Abbreviations: FCHV, female community health volunteer; IDI, In‐depth interviews; KII, key informant's interviews.

Judgmental sampling was used as a sampling method for selecting participants for KII and IDI. Among the participants of quantitative data, 15 Chepang mothers who were pregnant in their adolescence (10–19 years of age) were selected purposively for qualitative interviews. The key informants were chosen on the basis of their working experience among Chepangs, familiarity with their sociocultural dynamics, and willingness to give information. Participants who did not give consent to participate in the study, were seriously ill, or had communication difficulties due to speaking and hearing impairments were excluded from the study.

### 2.3 Data Collection

IDIs were done with the mothers using an interview guide, and KIIs were done with the health workers and officers using a separate KII guide. The IDI guide and KII guide were developed from an extensive literature review and discussion with the subject matter experts. The interview guides have been provided as a Supporting Information section (see Supporting Information File 1). The time duration for the qualitative interviews was 20–45 min, depending on the participant's response. The interviews were conducted by the principal investigator. To ensure cultural sensitivity and linguistic accuracy, the interview guides were initially developed in English and subsequently translated into Nepali.

### 2.4 Data Processing and Analysis

For the qualitative study, the recorded interviews were transferred to the computer and then first transcribed and translated into the English language as soon as possible after the interview was over. All transcripts were anonymized before data analysis by assigning respondent IDs such as A1, A2, …, An for IDIs with adolescent mothers, and K1, K2, …, Kn for interviews with key informants. The information from the interviews (IDIs and KIIs) was converted to “txt” format and then imported into RQDA (an R package for qualitative data analysis) software [17]. Each transcript was read and again re‐read to find a similar kind of response. It was then marked, and different codes were generated. This procedure was repeated for all transcripts.

Braun and Clark's six‐step thematic analysis with inductive coding was used to perform the qualitative analysis [18]. The themes, subthemes, and codes identified to explore determinants of adolescent pregnancy are presented in Table 2.

**TABLE 2:** Characteristics of Chepang mothers who participated in in‐depth interviews.

Code in the transcripts	Code in manuscript	Age (completed years)	Age at first pregnancy	Years of education	Place of residence
IDI1	A1	22	14	0	Ward 5
IDI2	A2	18	16	5	Ward 8
IDI3	A3	23	18	12	Ward 5
IDI4	A4	25	15	4	Ward 5
IDI5	A5	24	16	5	Ward 5
IDI6	A6	23	17	6	Ward 8
IDI7	A7	24	16	3	Ward 8
IDI8	A8	21	17	4	Ward 5
IDI9	A9	20	16	5	Ward 8
IDI10	A10	17	17	7	Ward 8

Abbreviation: IDI, In‐depth interviews.

### 2.5 Trustworthiness of the Study

The trustworthiness of this study was ensured by using Guba's constructs of rigor [19]. Credibility was maintained by developing the interview guide through an extensive literature review and expert input. Iterative questioning was used, and all interviews were audio‐recorded. Member checking with three participants confirmed the findings. Dependability was ensured through inter‐coder reliability, with 58 codes matched between two researchers, resulting in a 76.51% agreement. Transferability was supported by providing detailed background information and detailed description of the research process, allowing for comparison and replication in similar settings. Confirmability was maintained by documenting the researcher's beliefs and experiences. A reflexivity statement is included in Supporting Information File 2.

## 3 Results

### 3.1 Characteristics of Participants

Ten IDIs were conducted with mothers who experienced their first pregnancy during adolescence. (Table 2) Likewise, a total of 10 KIIs were conducted (Table 3).

**TABLE 3:** Characteristics of the key informants who participated in the qualitative study.

Code in the transcripts	Code in manuscript	Age (completed years)	Sanctioned post	Period of serving
KII1	K1	22	Teacher	3 years
KII2	K2	52	FCHV	13 years
KII3	K3	31	FCHV	15 years
KII4	K4	42	FCHV	13 years
KII5	K5	30	FCHV	8 years
KII6	K6	28	Health coordinator	7 years
KII7	K7	45	Ward representative	7 months
KII8	K8	52	Ward representative	7 months
KII9	K9	33	Auxiliary health worker (AHW)	7 years
KII10	K10	46	Auxiliary nurse and midwife (ANM)	26 years

Abbreviations: FCHV, female community health volunteer; KII, key informant's interviews.

### 3.2 Themes

From qualitative analysis, six main themes emerged for determinants of adolescent pregnancy: (i) inadequate access and use of sexual and reproductive health services, (ii) gaps in programmatic implementation, (iii) elopement marriage as a thoughtful escape, (iv) discontinuation of education, (v) limited knowledge and understanding of marriage, pregnancy, and childbirth, and (vi) early pregnancy is a sign of prosperity. (Table 4)

**TABLE 4:** Themes, subthemes, and codes identified to explore determinants of adolescent pregnancy.

Theme	Subthemes	Codes
1. Inadequate access and use of sexual and reproductive health services	Lack of access to sexual and reproductive health services	Distance to the health facility, out‐of‐pocket health expenditure, geographical constraints, seasonal difficulty, fear and shy to visit health institutions, unavailability of family planning device
	Health barriers to using contraceptive methods	Health problems after using contraception, fear of not having a child after using contraception
	Contraception is the sole responsibility of women	Women mostly use contraceptives, rare use of condoms, husbands disagreement with using the family planning method
2. Gaps in programmatic implementation	Programs and Policies to reduce child marriage and adolescent pregnancy	Lack of relay of information, incentives provision, ineffectiveness of programs, lack of legal action, monetary compensation
	Role of various stakeholders	Role of FCHV, counseling, role of external agencies, role of leaders, collaborative efforts, role of local government
3. Elopement marriages as a thoughtful escape	Elopement is the new trend	Falling in love, willingness to marry, elopement is a new trend, marriage against parent's will, fear of not marrying their chosen partner
	Repercussion of media and technology	Access to media and technology, social media is the cause of elopement
	Immaturity and unfulfilled desires	Unfulfilled desires, household work burden, immaturity
4. Discontinuation of education	Hurdles to access secondary‐level education	Lack of secondary schools, scholarship and allowances program, schooling after marriage is stigma, fear of society
	Apathy toward learning	Low interest in studies, language barrier, getting spoiled
	Poverty‐induced dropout from schools	Financial crisis, school dropout, unemployment, no habit of saving, child labor, work at an early age
5. Limited knowledge and understanding of marriage, pregnancy, and childbirth	Limited knowledge and understanding of contraception	Lack of knowledge on use of FP, abortion better than contraceptive use, being weak after sterilization
	Limited knowledge and understanding of adolescent pregnancy and childbirth	Giving birth at a young age is beneficial, perception regarding pregnancy and childbirth, lack of sex education
	Unplanned pregnancy	Not knowing about signs of being pregnant, lack of awareness
6. Early pregnancy is a sign of prosperity	Norms regarding marriage and pregnancy	Early marriage, husband age at marriage, sister age at marriage, religious values and norms, mother age at pregnancy, reluctance to change
	Societal, family, and peer pressure	Family pressure to have a child, peer influence, persuasion to marry, societal pressure, lack of parental guidance, negligence from parents
	Women's position in society	Son preference, lack of support from husband, giving birth is the reason for existence, violence against women

Abbreviation: FCHV, female community health volunteer.

#### 3.2.1 Inadequate Access and Use of Sexual and Reproductive Health Services

A recurring theme among participants was the difficult journey they had to undertake in order to reach health institutions, often requiring long walks spanning several hours. Furthermore, the absence of ambulance or transportation services worsened the difficulties faced in reaching these healthcare facilities. Regarding use, although participants demonstrated increasing awareness and willingness to utilize contraception, health‐related barriers often prevented them from doing so. A Chepang mother shared her negative experience with contraceptive use:

Depo caused heavy bleeding… I preferred giving birth to another child over enduring the pain of bleeding. (A1, 22 years old, Chepang Mother, Ward 5)

Furthermore, the burden of family planning and contraceptive use fell disproportionately on women within the Chepang community. This unequal distribution of responsibility is illustrated by the experience of a Chepang mother as she recounted her husband's reaction to her attempt to use condoms:

When my husband saw the oily substance inside, he immediately asked me to throw it away… and called me a prostitute. (A9, 20 years old, Chepang Mother, Ward 8)

This anecdote reveals not only male resistance to contraceptive use but also underlying misconceptions and mistrust surrounding family planning.

#### 3.2.2 Gaps in Programmatic Implementation

While programmatic aspects played a crucial role in addressing adolescent pregnancy and early marriage within the Chepang community, implementation gaps limited their effectiveness. One noteworthy policy implemented by the rural municipality was the birth registration policy, which, coupled with a financial incentive, showed promise in reducing these practices. As a health worker shared:

If someone gets married early then they cannot register the birth of their child and they also will not be able to get Rs. 5000 incentive given by the ward. (K9, 33 years old, AHW)

However, a critical gap in awareness about this provision within the community undermined its potential. As one health coordinator explained:

There is less information to the people about the incentive provision… those who are not socially active will not know about it. (K6, 28 years old, Health Coordinator)

The fact that only “socially active” individuals are aware of the incentive suggests that outreach efforts are not effectively reaching the broader community.

#### 3.2.3 Elopement Marriages as a Thoughtful Escape

In our study, an elopement marriage refers to a union where a couple marries without the formalities of legal registration, often without parental consent, and based solely on the individuals’ own will. Elopement marriages are culturally recognized but are not legally registered under civil law if the individuals are under 20 years old. Within the Chepang community, elopement marriages have become increasingly prevalent, replacing the more common practice of arranged marriages.

Previously, child marriage used to happen because of parent's pressure, but now they do it of their own will. (K5, 30 years old, FCHV)

This shift toward elopement marriages suggests a change in the dynamics of marriage within the community, with young people taking more control over their marital choices. The advent of media and communication technologies, such as mobile phones and social media platforms, has further facilitated these elopement marriages. An FCHV explained:

Every adolescent has access to mobile phones… They exchange numbers, connect on social media, fall in love, and run away. (K2, 52 years old, FCHV)

This highlights the significant role of technology in enabling adolescents to connect, form relationships, and ultimately elope. Beyond access to technology, participants noted that unfulfilled desires for better food and clothing, driven by poverty and difficult household situations, contribute to the prevalence of elopement marriages. These elopements can be seen as a “thoughtful escape” for Chepang adolescents seeking a different life, potentially free from parental pressures and the hardships of poverty. The lure of quick financial gains, though perhaps not always realistic, can also be a motivating factor.

#### 3.2.4 Discontinuation of Education

Access to secondary education is limited in this community, with only a few schools available, leaving students with limited or no options to continue their education beyond the primary level. The rainy season presented additional obstacles as rivers made it unsafe for students to cross and attend school. Tragically, there were instances where students were swept away by the strong currents of the river while attempting to reach school.

In addition, poverty‐induced dropout is prevalent, as families struggled to meet basic needs, including education expenses. One of the respondents shared her experience:

I was in 6th grade when I ran away… We didn't have enough money to purchase school uniforms or even food. (A5, 24 years old, Chepang Mother, Ward 5)

This illustrates the difficult choices families face and how poverty can force children, especially girls, to abandon their education. The cultural stigma attached to schooling after marriage further discourages married women from pursuing further education, limiting their opportunities for personal growth and economic empowerment. Additionally, child labor remains a significant barrier. A participant shared:

I studied up to grade two… I wanted to study. But then my mother warned me that if I didn't go to work, she would hit me with sisnu [stinging nettle leaf]. (A4, 25 years old, Chepang Mother, Ward 5)

This stark example highlights the harsh realities of child labor within the community and the pressures children face to contribute to the family income, often at the expense of their education. These intersecting factors—limited access, poverty, cultural stigma, and child labor—create a complex web of barriers that prevent many Chepang children from completing their education.

#### 3.2.5 Limited Knowledge and Understanding of Marriage, Pregnancy, and Childbirth

Misconceptions and fears surrounding contraception were prevalent, including concerns about infertility and uterine damage. A respondent shared a common belief:

I didn't know anything about contraception… everyone in the village used to say that if we use such contraceptives, we won't have children later. (A2, 18 years old, Chepang Mother, Ward 8)

Likewise, regarding the ideal age for childbirth, some participants believed that women over the age of 20 were considered too old to give birth as a participant expressed:

A woman at the age of 25 and 26 is already chippeko [considered old] to give birth… But if she is pregnant at the age of 15, then she can give birth. (A1, 22 years old, Chepang Mother, Ward 5)

This highlights the community's perception of early motherhood as ideal and the misconception that older women (even in their mid‐20s) are too old to give birth. Unplanned pregnancies were common, as many participants reported not realizing they were pregnant until several months into the pregnancy, indicating a lack of intention or family planning. A participant recounted her experience:

I didn't know pregnancy could stop my period, so I didn't find out until three months later when I started feeling sick. (A10, 17 years old, Chepang Mother, Ward 8)

#### 3.2.6 Early Pregnancy Is a Sign of Prosperity

Societal pressure to have a child at a young age adds to the complexity of the issue, with broader cultural and social norms influencing an individual's decisions to start a family early in life. In addition, the stigma associated with not having a child after marriage was significant in the community, leading to mistreatment by family and society. A participant shared her painful experience as

My husband would punish me… saying it was my fault for not being able to have a baby… he would beat me whenever I had a miscarriage. (A8, 21 years old, Chepang Mother, Ward 5)

This underscores the immense pressure women face to bear children and the severe consequences they may experience if they are unable to do so. Furthermore, within the Chepang community, having many children is often viewed as a sign of wealth and prosperity. Additionally, traditional gender roles and expectations place pressure on women to prioritize bearing children and tending to their families over their aspirations. Consequently, they may feel compelled to become pregnant at a young age due to the perception that they should have many children later in life.

## 4 Discussion

This study on the determinants of adolescent pregnancy among the Chepang ethnic group in Nepal has contributed to understanding the multifaceted factors influencing this issue. Our study found that the lack of access to sexual and reproductive health services served as a determinant of adolescent pregnancy, with one of the reasons being hesitance with service providers. Although Nepal National Adolescent Development and Health Strategy 2018 aimed to transform health facilities into adolescent‐friendly spaces [20], our research suggests a disconnection between policy and practice. Other studies in Nepal have also revealed a gap in Adolescent‐Friendly Health Services (AFHSs) utilization due to factors such as limited awareness, cost, satisfaction, and waiting room availability [21, 22]. Respecting the confidentiality of young people has been shown to effectively prevent adolescent pregnancy by the evidence [23].

Child marriage is rampant in the Chepang community and is a major reason for adolescent pregnancy. Although efforts have been made to reduce child marriage through legal means, these efforts are often ineffective. A key reason for this is that child marriages are not automatically void but only “voidable” once individuals reach 20 years of age [24]. This means that if a girl is married before 20 and has children, she cannot legally end the marriage until she reaches 20, effectively trapping her in the marriage. Given the social and cultural pressures on girls to prove their fertility, many girls become pregnant before they can seek legal recourse [25].

Registration of vital statistics such as births and marriages plays a critical role in preventing child marriage [26]. Although birth and marriage registration are compulsory in Nepal, the practice is often neglected in reality [27]. Consequently, many adolescents are unable to prove their actual age at the time of marriage, undermining the enforcement of laws prohibiting child marriage. This situation is perpetuated by low public awareness and inadequate monitoring of registration practices [25]. Moreover, existing policies requiring adolescents to wait until they are 20 years old to register their births or marriages present significant barriers. This delay prevents them from accessing essential government benefits, such as the 5000‐rupee allowance for delivering in health institutions and educational scholarships. Although these policies aim to curb adolescent pregnancies and child marriages, they inadvertently restrict adolescents’ rights to sexual and reproductive health services, as outlined in The Right to Safe Motherhood and Reproductive Health Act, 2018 [28].

A significant factor contributing to adolescent pregnancy in the Chepang community is the high dropout rate and discontinuation of education. Dropout is typically a process affected by various factors rather than a single cause [29]. Poverty plays a major role in this, as it not only makes it hard for families to cover school fees and related expenses but also increases the opportunity cost of education [30]. Additionally, options for secondary education are limited for Chepangs, with secondary schools often located at a distance. The availability of secondary school options significantly affects parental decisions regarding their children's continuation in primary education [31]. Further, in both Nepalese culture at large and among the Chepang community specifically, it is customary for women to leave school upon marriage or pregnancy to focus on managing their new household and caring for children [32].

Our study found that marriage by elopement was a common occurrence among adolescent pregnancies. Many adolescent girls enter marriage to avoid forced or arranged marriages or to escape challenging home situations, with some opting to elope due to societal norms that discourage sexual expression outside of wedlock [33]. Within the context of our study, it is important to note that the Chepang community, belonging to the Tibeto‐Burman ethnic group, has unique cultural practices. These practices, which share similarities with Western culture, include a notable emphasis on young individuals having a significant role in choosing their spouses [34]. The increase in love marriages has also been commonly associated with factors like educational expansion, technological advancements, and foreign influences, alongside broader socioeconomic shifts [35].

In our study, it was discovered that male partners who were abusive displayed reluctance toward using family planning methods, including condoms. This finding aligns with another study conducted among sexually active adolescent females, where it was reported that abusive male partners often had an interest in impregnating their partners at a young age [36]. Perceptions of masculinity strongly influence young men's decisions regarding sexual activity and contraception use, often driven by a desire to conform to peer expectations [37, 38]. Furthermore, adolescent males frequently perceive birth control usage as solely the responsibility of their female partners, which shows a lack of concern for the potential consequences, such as adolescent pregnancy and parenthood [39]. Although our study did not explore the perceptions of partners of adolescent pregnant women, previous research findings indicate that adolescent boys were less likely than their female counterparts to report feelings of embarrassment about the prospect of adolescent pregnancy [40]. Future studies could incorporate this component to provide a more comprehensive understanding of the dynamics surrounding adolescent pregnancy.

In this study, the notion of having many children was viewed as a symbol of wealth and prosperity within the community. This inclination may be influenced by the perception that having multiple children contributes to their tribe, thereby increasing the community's population and supporting the tribal group [41]. Cultural gender norms and beliefs, including the high social value placed on motherhood and marriage, contribute to views against contraception, often encouraging early pregnancy and marriage aspirations among girls, as well as leading to non‐use of contraception [42].

Our study has certain limitations. The use of judgmental sampling may have introduced selection bias, as the selected participants may have unique experiences or perspectives that were not shared by others in the larger population. The use of intercoder percentage agreement and participant checking reduced the risk of researcher bias. However, the subjectivity may not have been avoided completely during qualitative data analysis and interpretation.

## 5 Conclusion

In conclusion, the analysis revealed six main themes for determinants of adolescent pregnancy within the Chepang community in Raksirang. The findings highlighted the challenges faced by adolescents in accessing reproductive health services, the lack of targeted programs and awareness campaigns, the rise of elopement marriages and child marriages, limited educational opportunities, the lack of knowledge about family planning methods, and the influence of socio‐cultural beliefs on early marriage and childbearing. In essence, a holistic and collaborative approach is required, bringing together education, healthcare services, awareness programs, and a shift in sociocultural norms to empower Chepang adolescents and reduce the prevalence of adolescent pregnancy.

## Author Contributions

**Kusumsheela Bhatta**: conceptualization, methodology, writing – original draft preparation, writing – review and editing. **Pratiksha Pathak**: supervision, validation, project administration. **Madhusudan Subedi**: conceptualization, supervision, writing – review and editing.

## Ethics Statement

Approval of the study was taken from the Institutional Review Committee of Patan Academy of Health Sciences (IRC‐PAHS Ref: PHP2209061673.A1). Informed Written Consent was obtained from all participants's guardians for eligible participants less than 18 years before the interview. Guardianship typically involved the husband if of legal age or their parents‐in‐law in cases where both spouses were minors. Assent was taken from adolescents below 18 years after obtaining informed consent from the guardian. Informed consent was taken from eligible participants above 18 years of age. Confidentiality of the information was maintained and their personal identifying number was not disclosed.

## Conflicts of Interest

The authors declare no conflicts of interest.

## Supporting information



Supporting Information

Supporting Information

## Data Availability

Data will be made available on request.
