# Supplementary material for: Understanding the Determinants of Adolescent Pregnancy among an Indigenous Community in Rural Nepal: A Qualitative Exploration
Source: Public Health Chall. 2025 Mar 11;4(1):e70041. doi: 10.1002/puh2.70041 (PMC12039362; doi:10.1002/puh2.70041)
Supplement: Supplementary file 1 — Supporting Information [file PUH2-4-e70041-s001.docx]

**Reflexivity**

In the course of conducting qualitative research, the acknowledgment of the role played in the research process, including past experiences, assumptions, and beliefs that may have influenced the study, is imperative. Throughout this research project, transparency regarding the subjective perspective of the qualitative research team has been maintained. Efforts have been made to embrace and articulate personal perspectives throughout the study.

Given the unfamiliarity of the researchers with the Chepang community, support was sought from local non-governmental organizations with over five years of experience in the study area. Effective communication and collaboration with these organizations proved invaluable, particularly in establishing early relationships with locals, female community health volunteers, and health workers.

To ensure trust and acceptance within the local community, assistance was sought from individuals familiar with the customs and traditions of the Chepang community. The researcher had the companionship of two Chepang sisters throughout the data collection process, whose guidance was instrumental in establishing rapport with community members and overcoming language barriers. The researcher was grateful to learn from them the customary greeting, "Jay Masi", which is highly preferred by the Chepang community and helped me to assimilate better into their culture.

In the field, the researcher quickly realized that their youthful appearance had not been anticipated by the locals based on previous phone conversations. However, this turned out to be advantageous, particularly given that the respondents were less than 25 years old. The relatable age of the researcher and the respondents facilitated a deeper level of trust, fostering an environment where participants felt comfortable sharing personal information, including reproductive health experiences. This led to the establishment of a deeper level of trust, and a sense of being regarded more as a friend or big sister emerged, someone present to listen and offer support in their struggles and joys.

During the time in the field, concerns were initially held regarding the potential for participants to feel pressured into conforming to desired responses or adjusting their answers based on the researcher's education and social standing. However, it was found, to the researcher's delight, that participants remained steadfast in their beliefs and values. Responses were characterized by honesty and genuineness, with no apparent influence from the researcher's background or credentials. The sincerity of participants was greatly appreciated, and the researcher was impressed by their unwavering commitment to their own belief system.

While conducting qualitative interviews, ethical considerations and potential power dynamics were consistently borne in mind. A conscious effort was made to foster trust and rapport with participants, creating an environment conducive to sharing their experiences comfortably. At times, the researcher unintentionally shifted from the role of a mere observer to engaging in heartfelt conversations with participants. To prioritize their comfort, the decision was made to abstain from recording these conversations, with a focus on active listening and empathy. The paramount goal was to ensure that participants felt heard and understood, and the researcher expressed gratitude for the opportunity to establish a personal connection with them.

Some of their life stories would even cause emotional distress. Emotional lows were experienced, and efforts were made to seek ways to provide support. An instance arose where guidance was requested by a respondent, aged 16 and having recently given birth, on obtaining an abortion pill. Information on where to obtain one was provided, along with education on family planning methods, a discussion initiated after the data collection. Among the respondents, some as young as 15 were contemplating home delivery, while others lacked awareness of family planning and ANC services. As part of their ethical duty, comprehensive information on these matters was provided to all participants after the conclusion of the data collection process.

As a public health professional, the initial motivation was guided by the belief that adolescent pregnancy inherently led to negative health consequences. However, upon entering the field, it was realized that the community's experiences differed significantly from initial expectations. The initial beliefs were challenged, and an effort was made to comprehend their perspectives and worldview, approaching questions with a neutral mindset. Consequently, there was a heightened enthusiasm and eagerness to learn about their ways of life, thinking, and attitudes toward health.

During the interviews, the focus was on listening to their values without imposing any preconceived notions. Cherished moments included living life as they did—enjoying their food, participating in their festivals (chewar), dancing with them, and attending church on Saturdays. These experiences facilitated a deeper understanding beyond what could be gleaned from the 20-45 minute interviews, revealing the beauty of being a part of qualitative research.

Being an outsider in the community was found to be beneficial during the data collection process. The absence of prior connections or history with the locals contributed to their increased comfort in sharing information, reassured by the guarantee of confidentiality.

The interviews were meticulously conducted in private and comfortable settings for the participants. Frequently, visits were made to nearby fields where no one was present to further ensure privacy and confidentiality. Additionally, to enhance participant ease, interviews were conducted without the presence of local assistants who were native to the community. This approach allowed respondents to openly and freely share their experiences and thoughts.

The disclosure of income status was an aspect participants seemed hesitant to share. It was apparent that a significant segment of the community was involved in weed and opium farming, activities deemed illegal in Nepal. Understandably, there was fear that discussing their engagement in such practices could lead to police intervention and the destruction of their crops, which often served as their primary source of income. Consequently, participants refrained from disclosing their earnings or revenue generated from the sale of these products during the interviews.

Gathering data from the community proved to be a challenging yet fulfilling experience, given the geographical hurdles that had to be surmounted. The entire ward was susceptible to landslides, adding complexity to the task. Many participants were occupied with farming activities, necessitating efforts to locate them in the fields. This often involved climbing steep hills and crossing rivers multiple times. While physically demanding, the experience gained from hiking and trekking proved beneficial, and the breathtaking views from the hilltops made the exertion worthwhile. A profound fondness for both the place and its people developed. Fortunately, favorable weather conditions facilitated more manageable travel and walking.

The observation of many members of the Chepang community converting from Hinduism to Christianity, leading to abstinence from alcohol, was intriguing. However, due to the physical demands of fieldwork, a few individuals still engaged in alcohol consumption. During data collection, some respondents were under the influence of alcohol, necessitating the repetition of questions to ensure their full understanding. Despite this challenge, a stance of respect and patience was maintained throughout the interviews.

A strategic decision was made to initiate data collection in Ward 5, where access and transport were comparatively more straightforward, before progressing to the more demanding terrain of Ward 8. In hindsight, this decision proved to be judicious, facilitating a smoother transition and adaptation to the new environment.

Being a Hindu researcher working in a community predominantly converted to Christianity presented a new and unique experience. Pastors in the community frequently shared their perspectives on Christianity, Jesus, and the advantages of following their religion. While deeply respecting their values and beliefs, there was a need to remain mindful of potential influence or manipulation. This situation provided an opportunity to broaden understanding of different religions and cultures, emphasizing the importance of maintaining a neutral perspective during the data collection process.

Communicating to respondents that the research study did not offer immediate benefits proved to be a significant challenge. The community had grown accustomed to receiving assistance and support from outsiders, either individually or through institutions.

Despite facing challenges of poverty and limited access to basic necessities, the community warmly welcomed the researcher with open arms. Locals went out of their way to provide the best available food and ensure safety and comfort. However, occasional conversations in the Chepang language, which the researcher did not understand, made them feel like an outsider. Laughing along without fully grasping the context served as a valuable lesson in the importance of knowing the language of the community being worked with.

In addition, the participants and the entire community expressed gratitude for the researcher's presence despite the challenging living conditions such as difficult terrain, lack of electricity, proper food and accommodation, and even basic necessities. Appreciation was voiced by everyone encountered on the journey for the efforts made to travel, listen to them, and conduct a study on their health status. This, it is believed, instilled in them a sense of significance and the feeling of being appreciated.

On the final day of data collection, emotions welled up as it was recognized that it was time to depart from the community that had become closely attached to. Throughout the stay, interactions with numerous individuals, observations of their culture, and the development of a profound connection were integral aspects. The warm and welcoming nature of the community, their openness to share feelings, and their innocent values left an indelible mark on the heart. Gratitude is extended for the time and effort invested by all participants in the study and their willingness to share their experiences.

Reflecting on the time spent in the community, the researcher feels humbled and honored to have had the opportunity to conduct research with the potential to impact health outcomes for pregnant adolescents. The hope is that the evidence gathered will contribute to targeted health interventions, preventing complications during antepartum, childbirth, and postpartum periods. Lastly, the researcher looks forward to future collaboration with the community and expresses gratitude for the lasting memories and experiences gained.
