# Supplementary material for: Understanding the Determinants of Adolescent Pregnancy among an Indigenous Community in Rural Nepal: A Qualitative Exploration
Source: Public Health Chall. 2025 Mar 11;4(1):e70041. doi: 10.1002/puh2.70041 (PMC12039362; doi:10.1002/puh2.70041)
Supplement: Supplementary file 2 — Supporting Information [file PUH2-4-e70041-s002.docx]

Qualitative In-depth Interview Guide -English

**In-depth Interview Guideline with Adolescent Mothers**

| **Basic Information** | | | | |
| --- | --- | --- | --- | --- |
| Consent Given |  | Yes |  | No |
| Date (dd/mm/yy): |  | | | |
| Age (completed years): |  | | | |
| Age at first pregnancy: |  | | | |
| Education Level: |  | | | |
| Place of residence: |  | | | |
| Interview start time: |  | | | |
| Interview end time: |  | | | |
| Respondent ID: |  | | | |

| **Topic** | **Main Question** | **Follow up Question** | **Probes** |
| --- | --- | --- | --- |
| Marriage | How did you come to the decision of marriage at the age of….? | Did you decide to get married on your own? |  |
|  |  | If yes, what was the reason behind the decision? | Education level, Societal norms, socioeconomic status, relationship status |
|  |  | If no, how was the decision made? |  |
|  |  | Were you happy with it? |  |
| Family norms | Can you tell me about the pregnancy and childbirth history/norms that are existing in your family? | At what age did your family members (mothers, in-laws, others) get pregnant for the first time? |  |
|  |  | What factors do you think influenced their decision to get pregnant at that age? |  |
|  |  | How many children did they have? |  |
|  |  | What changes have occurred in pregnancy and child birth norms across generations in your family? | Number of children, Birth spacing, Age at pregnancy |
| Factors affecting teenage pregnancy | What led you to the decision of having a baby at the age of ..? | What influences did your family and peer play in your decision? | Counseling, Pressure |
|  |  | What role did you and your husband had in the decision-making? | The decision about being pregnant, Decision on using contraception  Dominant role in decision making |
|  |  | What factors played role in your pregnancy? | Education, School availability, Drop Out, Income, Occupation, Livelihood, Women’s status, Substance use, media exposure |
| Family Planning | What do you know about family planning services and contraceptives? | What means of contraception did you or your partner use? |  |
|  |  | Why did you use particular means of contraception? |  |
|  |  | If no, why didn’t you use any means of contraception? |  |
|  |  | In your community, where can teen mothers like yourselves go to get family planning services and information? | Health institution, school, FCHV |
|  |  | What barriers are there to seeking family planning services or information? |  |
|  |  | If today you wanted to get information on family planning where would you go and why? | What information or services can you get there? |
| Perception | What do you think is the right age to have a child? | Why do you think so? |  |
|  |  | What do you think are the consequences of being a mother at a young age? |  |
| Societal norms | What societal norms exist regarding teenage pregnancy and childbirth in your community? | What norms exist regarding preference for the gender of a child? |  |
|  |  | How happy are you with the norms of society regarding teenage pregnancy? |  |
| Closing | Is there anything else that we didn’t discuss that you think is important to know when discussing determinants of teenage pregnancy? | | |

**THANK YOU FOR YOUR TIME!**

Qualitative KII Guide -English

**Key-Informant Interview Guideline**

| **Background Information** | | | | | | | | | |
| --- | --- | --- | --- | --- | --- | --- | --- | --- | --- |
| Consent Given |  | Yes | | | |  | | No | |
| Date (dd/mm/yy): | Day | |  | Month |  | | Year | |  |
| Interview start time: |  | | | | | | | | |
| Interview end time: |  | | | | | | | | |
| Respondent ID: |  | | | | | | | | |
| Age (completed years): |  | | | | | | | | |
| Working ward and facility: |  | | | | | | | | |
| Sanctioned Post: |  | | | | | | | | |
| Years of Serving: |  | | | | | | | | |

| **Main Question** | **Follow up Question** | **Probes** |
| --- | --- | --- |
| What is the status of child marriage in Chepang community? | Is the child marriage rate increasing or decreasing over time among Chepang community? | Why? |
|  | What factors cause child marriage in Chepang community? | Education level, Societal norms, socioeconomic status, relationship status |
|  | Who primarily makes marriage decisions in families among Chepang communities? |  |
| Can you tell me about the pregnancy and childbirth history/norms that exist in Chepang communities? | Is the teenage pregnancy prevalence in Chepang communities decreasing or increasing over time? | Why? |
|  | Have there been changes in pregnancy and childbirth norms across generations among Chepang communities? | Number of children, Birth spacing, Gender Preference |
| What do you think are the factors affecting teenage pregnancy among Chepang communities? | What family planning services exist in Chepang community? | Contraceptives, IEC, Counseling |
|  | Where can teen mothers go to get family planning services and information? |  |
|  | Are there any barriers to providing family planning services or information? | Attitude of Chepang community towards contraception |
|  | Do husband and wife have an equal role in the decision-making? | Decision about being pregnant, Decision on using contraception |
|  | What socio-economic and cultural factors affect teenage pregnancy in Chepang communities? | Education, School availability, Drop Out, Income, Occupation, Livelihood, Women’s status, Substance use, media exposure |
| What preventive measures or interventions are currently in place to address adolescent pregnancy in the Chepang community? | What efforts are there efforts to promote gender equality and empower adolescents to make informed decisions about their reproductive health? | External development partners, laws, policies, role of government, role of health workers, national priority, budget |
|  | In your opinion, what strategies could be effective in reducing the prevalence of adolescent pregnancies in this community? |  |
|  | What community-driven initiatives or cultural practices could be leveraged to address the issue? |  |
| Would you like to tell me anything or suggest anything that you think I have left or is important? | | |

**THANK YOU FOR YOUR TIME!**
